# Intensive Longitudinal Social Sensing in Patients With Psychosis Spectrum Disorders: An Exploratory Pilot Study

**DOI:** 10.1093/schbul/sbae032

**Published:** 2024-03-24

**Authors:** Moritz von Heyden, Paul Grube, Markus Sack, Johannes Wiesner, Oliver Frank, Kathrin Becker, Stefan Heintz, Iris Reinhard, Sarah Hohmann, Dusan Hirjak, Andreas Meyer-Lindenberg, Urs Braun

**Affiliations:** Department of Psychiatry and Psychotherapy, Central Institute of Mental Health, University of Heidelberg, Medical Faculty Mannheim, Mannheim, Germany; Hector Institute for Artificial Intelligence in Psychiatry, Central Institute of Mental Health, University Heidelberg, Medical Faculty Mannheim, Mannheim, Germany; Department of Psychiatry and Psychotherapy, Central Institute of Mental Health, University of Heidelberg, Medical Faculty Mannheim, Mannheim, Germany; Hector Institute for Artificial Intelligence in Psychiatry, Central Institute of Mental Health, University Heidelberg, Medical Faculty Mannheim, Mannheim, Germany; Department of Neuroimaging, Central Institute of Mental Health, University Heidelberg, Medical Faculty Mannheim, Mannheim, Germany; Department of Psychiatry and Psychotherapy, Central Institute of Mental Health, University of Heidelberg, Medical Faculty Mannheim, Mannheim, Germany; Hector Institute for Artificial Intelligence in Psychiatry, Central Institute of Mental Health, University Heidelberg, Medical Faculty Mannheim, Mannheim, Germany; Department of Psychiatry and Psychotherapy, Central Institute of Mental Health, University of Heidelberg, Medical Faculty Mannheim, Mannheim, Germany; Hector Institute for Artificial Intelligence in Psychiatry, Central Institute of Mental Health, University Heidelberg, Medical Faculty Mannheim, Mannheim, Germany; Department of Child and Adolescent Psychiatry and Psychotherapy, Central Institute of Mental Health, University Heidelberg, Medical Faculty Mannheim, Mannheim, Germany; Department of Child and Adolescent Psychiatry and Psychotherapy, Central Institute of Mental Health, University Heidelberg, Medical Faculty Mannheim, Mannheim, Germany; Department of Biostatistics, Central Institute of Mental Health, University Heidelberg, Medical Faculty Mannheim, Mannheim, Germany; Department of Child and Adolescent Psychiatry and Psychotherapy, Central Institute of Mental Health, University Heidelberg, Medical Faculty Mannheim, Mannheim, Germany; Department of Child and Adolescent Psychiatry and Psychotherapy, University Medical Center Hamburg-Eppendorf, Hamburg, Germany; Department of Psychiatry and Psychotherapy, Central Institute of Mental Health, University of Heidelberg, Medical Faculty Mannheim, Mannheim, Germany; Department of Psychiatry and Psychotherapy, Central Institute of Mental Health, University of Heidelberg, Medical Faculty Mannheim, Mannheim, Germany; German Center for Mental Health, Partner site Mannheim-Heidelberg-Ulm; Department of Psychiatry and Psychotherapy, Central Institute of Mental Health, University of Heidelberg, Medical Faculty Mannheim, Mannheim, Germany; Hector Institute for Artificial Intelligence in Psychiatry, Central Institute of Mental Health, University Heidelberg, Medical Faculty Mannheim, Mannheim, Germany; German Center for Mental Health, Partner site Mannheim-Heidelberg-Ulm

**Keywords:** psychosis spectrum disorders, social interaction, RFID sensors, negative symptoms, schizophrenia, social

## Abstract

**Background:**

Psychosis spectrum disorders are characterized by significant alterations in social functioning, which is a major factor for patient recovery. Despite its importance, objectively quantifying the complex day-to-day social behavior in real-life settings has rarely been attempted. Here, we conducted a pilot study with wearable sensors that passively and continuously register interactions with other participants. We hypothesized that the amount and pattern of social interaction was associated with the severity of psychotic symptoms.

**Study Design:**

We recruited 7 patients with psychosis spectrum disorders and 18 team members from a Soteria-style ward. Each participant wore a radio frequency identification badge, sending and receiving signals from nearby badges, allowing passive quantification of social interactions. In addition, symptom severity was assessed weekly by the Positive and Negative Syndrome Scale (PANSS).

**Study Results:**

During an 11-week period, we identified 17 970 interactions among patients and staff. On average, patients spent 2.6 h per day interacting, capturing relevant aspects of daily social life. Relative daily interaction time, average interaction duration, and clustering coefficient, a measure of local network integration, were significantly associated with lower PANSS scores. Self-reported interaction time did not correlate with measured interaction time or with PANSS, indicating the importance of objective markers.

**Conclusions:**

This pilot study demonstrates the feasibility of passively recording social interaction of patients and staff at high resolution and for a long observation period in a real-life setting in a psychiatric department. We show links between quantified social interaction and psychopathology that may facilitate development and personalization of targeted treatments.

## Introduction

Social interaction deficits are one of the earliest signs of psychosis spectrum disorders (PSD) and do not respond well to antipsychotic medication.^1–4^ Furthermore, social functioning is one of the strongest predictor of long-term outcomes, affecting occupational functioning and quality of life.^4–9^ They are therefore a focus of treatment development, especially in psychotherapy.^10,11^ Trying to unravel the relationship between social functioning, psychotic symptoms, and functional outcomes, however, is challenging. In particular, a key obstacle is to quantify social functioning in ecologically valid ways that capture the complexity of real-life social interactions. So far, social functioning in PSD has been assessed using social-cognitive testing or rating scales,^6,7^ providing only limited and static insights into everyday life social behavior.

**Fig. 1.**
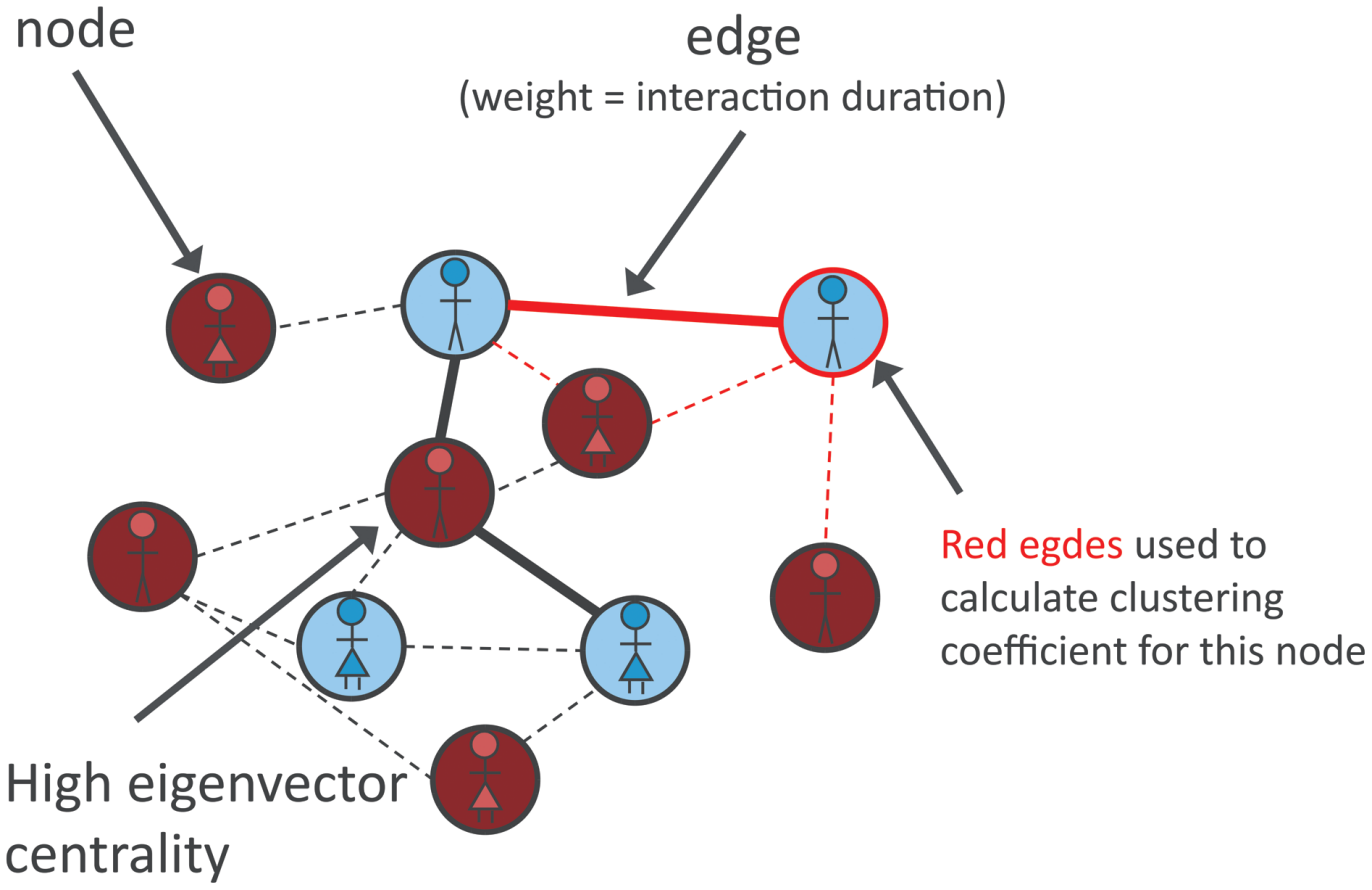
Creating a weighted network graph. A graph consists of nodes, connected by edges. These edges can be weighted based on observed data (eg, interaction duration). From such a graph, node properties can be calculated: eigenvector centrality is a measure of how easily a node can reach all other nodes via the available edges and can thus be understood as a measure of importance. The clustering coefficient measures the weight of the edges to as well as between neighboring nodes and can thus be understood as measure for how strongly a node clusters together with its neighbors.

Recent development in the technology of wearable sensors promise novel, unobtrusive, and objective ways to capture everyday behavior of patients.^12–15^ Radio frequency identification (RFID) badges have been used to successfully capture real-life social interactions among a selected group of individuals at high temporal resolution.^13–16^ These sensors show a high accuracy in reconstructing actual social interactions and allow the construction of social network graphs.^13–15,17^ In social network graphs, participants are represented as nodes and social ties as weighted edges between them (Figure 1). This can be expressed in an adjacency matrix, which allows to calculate node attributes. As opposed to the binary “interacting”/”not-interacting” derived from merely looking at one patient’s sensor log, node attributes derived from a network graph capture the relationship between a participant and the group and are able to describe the more complex aspects of social interaction in groups.

While sensor-based measures of social interaction in healthy populations have investigated the dynamics of social gatherings and the scale of social networks,^14,18^ studies applying these technologies to study mental health or patient populations have been scarce. A recent study in a sample of university students found a link between the quantity of social interaction measured by sensors to the level of depressive symptoms.^16^

Other studies even investigated graph theoretic measures in relation to mental health. Higher eigenvector centrality, a measure of the relative importance of a single node in a network, has been associated with a decreased likelihood of depression in a large population-based cohort.^19^ In a recent study employing RFID sensors in a sample of company employees, the clustering coefficient, describing the tendency of a node to form part of a cluster of nodes, was negatively associated with depressive symptoms.^20^

The present study was conducted in the Soteria of the Central Institute of Mental Health in Mannheim (CIMH). The term “Soteria,” which originates from the psychiatry-critical movement of the 1970s, translates to “well-being, preservation, salvation” in the field of psychiatry.^21,22^ Psychiatrist Loren Mosher (1933–2004) spearheaded the establishment of a community-like hospital in California, United States as an alternative therapeutic approach for individuals suffering from psychosis. There, schizophrenia patients were accompanied during their acute psychotic episodes. Continuing from this, the Swiss psychiatrist Luc Ciompi established a Soteria project in Bern, Switzerland in 1984, which is in existence at present. In Germany, more than 6 Soteria units have now been established.

The Soteria at CIMH provides capacities for up to 12 inpatients between 16 and 26 years of age. Patients in the Soteria can be treated at all stages of their psychosis, including acute psychotic episodes. The main goal is to offer adolescents and young adults with psychosis a community-based environment, intensive personal connections (“being-with”), and meaningful shared activities (“doing-with”).^21,22^ The therapeutic strategy of “being with” refers in particular to the human interaction between patients with psychosis and Soteria staff. It is also the empathetic and intensive accompaniment of patients during their acute psychotic episode by the Soteria staff. In contrast to traditional treatment on a ward, patients are simply required to participate in “being-with” others at the start of therapy in Soteria and initially, patients do not have to complete any tasks or attend any therapeutic sessions. This is intended to build up the therapeutic relationship and the patients acceptance of the illness. The “doing-with” approach means that Soteria patients cope with the demands of everyday life together. In contrast to highly structured traditional wards where the therapists organize the program, in the Soteria everything is discussed and planned together. Both the patients themselves and the Soteria team work together to tackle everyday activities and events (shopping, cooking, cleaning, leisure activities, psycho-education, dealing with symptoms, crisis management, etc.). This therapeutic approach should enable patients to (re)gain everyday social and daily living skills. In summary, the Soteria setting intentionally resembles real-life living conditions, such as shared-flat settings of young adolescents or assisted living conditions for more severely ill patients and emphasizes social interactions throughout, providing an ideal setting for our pilot study.

This pilot study had 2 main objectives: first, to passively quantify real-life social interactions on a psychiatric ward in both patients and therapists for the first time. Second, to test the hypothesis that a higher volume of social interactions will be related to decreased psychopathological symptoms.

## Methods

### Participants

Seven patients were recruited from the Soteria at CIMH (table 1). Patient inclusion criteria were age between 16 and 26, diagnosis of a PSD according to ICD-10 or clinical high risk of psychosis, and sufficient proficiency in the German language.

**Table 1.** Sample Characteristics, Initial Clinical Assessment, Social Sensing Measures

	Patients (*n* = 7) *M* (SD)(range) or *n* (%)	Staff (*n* = 18) *M* (SD)(range) or *n* (%)
Demographics		
Age	21.3 (2.4)	39.7 (12.0)
Female	3 (43%)	14 (78%)
Lower secondary school diploma	3 (43%)	—
Comprehensive secondary school diploma	2 (29%)	—
Technical college diploma	2 (29%)	—
Currently employed	3 (43%)	—
Diagnosis		
Paranoid schizophrenia (F20.0)	2 (29%)	—
Schizotypal disorder (F21.0)	1 (14%)	—
Acute polymorphic psychotic disorder (F23.0)	1 (14%)	—
Mental and behavioral disorders due to use of cannabinoids psychotic disorder (F12.5)	2 (29%)	—
Bipolar affective disorder, current episode manic with psychotic symptoms (F31.2)	1 (14%)	—
Current is first psychotic episode	5 (71%)	—
Clinical scales at study admission		
B-CATS (*z*-score)	−1.36 (0.92)	—
PANSS total	45 (15.9)	—
PSP	53 (15)(30;75)	—
PSP at study completion	63 (16)(30;85)	—
BDI-II	21 (8)(7;31)	
Olanzapine equivalents	12 (13)(0;42)	
Social sensing		
*n* interactions (total = 17 970)	8 419	14 978
Number of days with data	26 (13)	19 (13)
Hours recorded per day	8.6 (1.7)	5.3 (1.7)
Daily interaction time		
Overall	2.6 (0.6)	2.5 (0.7)
With staff	1.1 (0.5)	1.9 (0.8)
With patients	1.9 (0.4)	0.8 (0.4)

*Note*: B-CATS, Brief Cognitive Assessment Tool for Schizophrenia; PANSS, Positive and Negative Syndrome Scale; PSP, Personal and Social Performance scale; BDI-II, Beck Depression Inventory-II.

Additionally, 18 members of staff participated in the study (table 1). Staff inclusion criteria were age between 18 and 65 and having their principal work assignment on the Soteria ward.

Exclusion criterion for all participants was the inability to give informed consent. To be able to record patient-patient interactions, we set a minimum of 3 patients participating simultaneously.

### Study Design

We conducted a longitudinal cohort study, continuously recording social interactions during an observation period from December 6, 2021 until February 18, 2022. Participants wore sensors during weekdays and between 8 am and 8 pm. Additionally, we performed clinical interviews and issued questionnaires weekly.

### Setting

Data were collected on the Soteria of the CIMH. The Soteria is an open unit focused on the treatment of adolescents aged 16–26 with early psychosis. The Soteria is designed to resemble a shared apartment, including large community spaces such as a kitchen, dining area, and couch area. Patients and staff handle everyday tasks, such as shopping and cooking, together, with the only mandatory activity being the shared meals (~1.5 h/day).

### Clinical Interviews

Once a week, structured clinical interviews were conducted with patients, and additional information was collected from staff to assess the Positive and Negative Syndrome Scale (PANSS) score. Upon entering and upon completing the study, the personal and social performance scale (PSP) was assessed based on an interview and information from staff.

### Questionnaires and Cognitive Assessment

Upon entering the study, patients underwent the Brief Cognitive Assessment Tool for Schizophrenia (B-CATS) assessment and were asked to complete a demographic questionnaire.

Patients were asked weekly to rate how much time they spent alone in their room (item 1), and how much of their leisure time they spent with others outside of their room (item 2) on a 7-point Likert scale. Patients were instructed to only rate the time that they spent on the ward and to omit weekends at home. The mean of item 1 (reverse-coded) and item 2 was taken as the index of self-reported interaction quantity. We asked patients weekly to answer the Beck’s Depression Inventory-II (BDI-II) questionnaire.

### Sensors

**Fig. 2.**
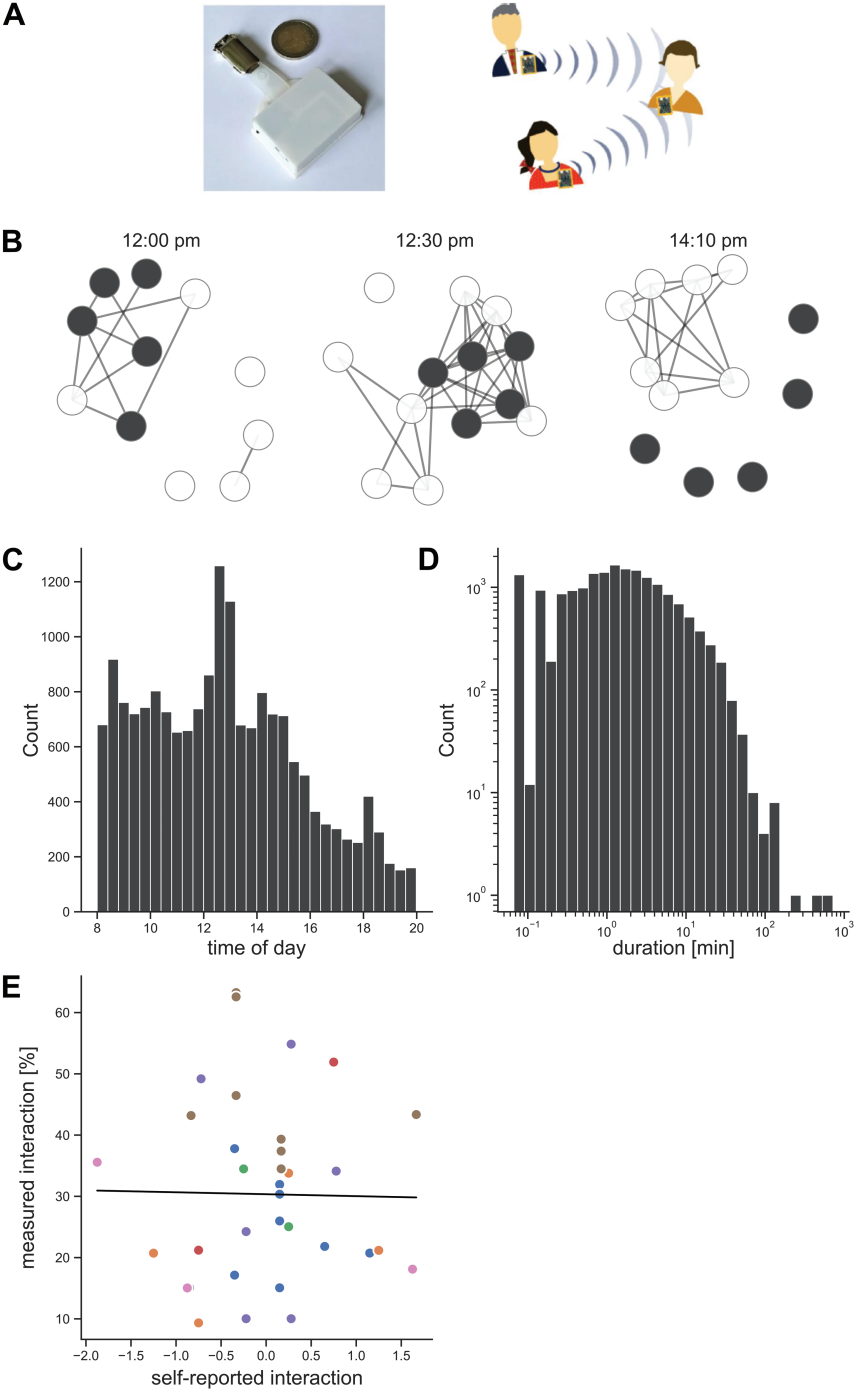
Properties of face-to-face interaction measured with passive sensors. (A) Schematic of the sensor set-up. (B) Representative snapshots of detected signals between participants at different times on 1 day. Patients (dark), staff (light). 12:00 pm: meal preparation, 12:30 pm: lunch, and 14:10 pm: team meeting. (C) Histogram of the beginning times of all interactions. (D) Histogram of all interaction durations on a log-log scale. (E) Mixed linear model with the self-reported interaction index as predictor and measured relative social interaction as the outcome.

For estimating contacts between participants, we used custom-built sensors, which registered contacts with other sensors if they were physically close to each other. Inspired by the Open Beacon Project,^23^ we combined an Adafruit *ItsyBitsy nRF52* board with a battery pack and stored it in a 3D-printed case to form a badge that could be worn on top of clothing (figure 2A). To synchronize the time between the devices we used a stationary microcontroller based on an *esp32* module, which is capable of Wi-Fi and Bluetooth 5.3 low energy (BLE). It was continuously connected to a Wi-Fi network and updated its internal date and time via Network Time Protocol servers. During startup, a badge automatically obtained the current date and time as a Unix timestamp from this device.

The badges were programmed to transmit 2.4 GHz radio signals (BLE). BLE advertising packages containing their own ID every 4 s, while continuously receiving incoming signals. Incoming signals from other badges were saved as sender ID, signal strength (RSSI: Received Signal Strength Indicator), and Unix timestamp on the internal memory of the receiving badge. Furthermore, every 5 min the badges recorded their battery status. As the badges work independently, they could also detect contacts taking place outside the ward. After recording, the data were transferred to a PC and stored.

### Data Processing

First, all duplicate data, as well as data from weekends and times between 8 pm and 8 am were removed. Signals with a RSSI value lower than −75 were filtered out, as previous test runs have determined this threshold to be an optimal balance between false positive and detection accuracy (supplementary figure 1). Based on the settings tested, this threshold corresponds to a distance of approximately 2 m (supplementary figure 1). A social interaction between 2 participants was defined as the period in which both badges continuously detected each other. Data were interpolated for gaps of up to 75 s, which was demonstrated by Elmer et al.^15^ to increase accuracy. In cases of data loss due to technical problems, no double-check of the contacts with this device was performed. For each participant, beginning, end, and duration of all interactions were calculated. The time a badge was active on a given day was defined as the summed inter-event intervals of the battery signal.

For further analysis, we computed the relative interaction time, the eigenvector centrality, and the weighted clustering coefficient for each day and then considered the mean values across the 6 days preceding and the day of each PANSS assessment. If there was an overlap, eg, less than 7 days between consecutive PANSS assessments, data were only used once for the respective closest PANSS assessment. The relative interaction time was defined as the total time a patient spent in social interaction on 1 day divided by the time the badge was active. We also computed the mean duration of a single interaction as the average of the common logarithm of the durations of all interactions a participant had during that time period. We used the logarithmic transformation because the durations exhibited a long-tailed distribution.

Weighted undirected graphs were constructed for each day using the NetworkX Python package^24^ (figure 1). A graph consists of nodes connected by edges. These edges can be weighted based on observed data (eg, interaction duration). From such a graph, node properties can be calculated: eigenvector centrality measures of how easily a node can reach all other nodes via the available edges and can thus be understood as a measure of importance. The clustering coefficient measures the weight of the edges to and between neighboring nodes and can thus be understood as a measure for how strongly a node clusters together with its neighbors (figure 1). Participants wearing a badge on that day were added as nodes. Interaction durations between 2 participants were summed together, and if the cumulative interaction duration across the day was longer than 10 min, the common logarithm of the total interaction duration was added as the edge weight between these 2 nodes. The cutoff was chosen in order to decrease the number of potentially noisy edges. Significantly, varying the cutoff values only marginally changed the results of the affected network graph parameters (eigenvector centrality and clustering coefficient) (supplementary figure 2). Weighted eigenvector centrality, introduced by Bonacich^25^ was computed for each node *i* as the *i* th element of the vector *x*:


Ax=λx (1)

where *A* is the adjacency matrix and λ its largest eigenvalue.

The clustering coefficient cu was computed for each node i, following the approach by Saramäki^26^:


$$c_{i}=\frac{1}{\text{deg(}i\text{) (deg(}i\text{)}-\text{1)})}\sum_{jk}{\left({\hat{w}}_{ij}{\hat{w}}_{ik}{\hat{w}}_{vjk}\right)}^{1/3}$$ (2)

where the edge weights are normalized by the maximum weight in the network:


$${\hat{w}}_{ij}=\frac{w_{ij}}{\max(w)}$$ (3)

### Statistical Analysis

We estimated linear mixed models in R and RStudio^27^ with the “lmer” R package^28^ to check for a linear relationship between time and parameters of social interaction, as well as to investigate if the weekly PANSS total score could be predicted from parameters of social interaction during the preceding week. In all models, random intercepts were estimated with observations nested within participants *i*. All predictors were group-mean centered.

Model 1: $y_{ij}=\beta_{0}+\beta_{1}\;{\text{SelfReportedInteraction}}_{j}+\alpha_{0i}+\epsilon_{ij},y:\text{RelativeSocialInteraction}$

Model 2: $y_{ij}=\beta_{0}+\beta_{1}{\text{Time}}_{j}+\alpha_{0i}+\epsilon_{ij},y:\text{RelativeSocialInteraction}$

Model 3: $y_{ij}=\beta_{0}+\beta_{1}{\text{Time}}_{j}+\alpha_{0i}+\epsilon_{ij},y:\text{InteractionDuration}$

Model 4: $y_{ij}=\beta_{0}+\beta_{1}{\text{Time}}_{j}+\alpha_{0i}+\epsilon_{ij},y:\text{Centrality}$

Model 5: $y_{ij}=\beta_{0}+\beta_{1}{\text{Time}}_{j}+\alpha_{0i}+\epsilon_{ij},y:\text{Clustering}$

Model 6: $y_{ij}=\beta_{0}+\beta_{1}{\text{RelativeSocialInteraction}}_{j}+\alpha_{0i}+\epsilon_{ij},y:\text{PANSSTotal}$

Model 7: $y_{ij}=\beta_{0}+\beta_{1}{\text{InteractionDuration}}_{j}+\alpha_{0i}+\epsilon_{ij},y:\text{PANSSTotal}$

Model 8: $y_{ij}=\beta_{0}+\beta_{1}{\text{Centrality}}_{j}+\alpha_{0i}+\epsilon_{ij},y:\text{PANSSTotal}$

Model 9: $y_{ij}=\beta_{0}+\beta_{1}{\text{Clustering}}_{j}+\alpha_{0i}+\epsilon_{ij},y:\text{PANSSTotal}$

Model 10: $y_{ij}=\beta_{0}+\beta_{1}{\text{SelfReportedInteraction}}_{j}+\alpha_{0i}+\epsilon_{ij},y:\text{PANSSTotal}$

*F*-tests of fixed effects were performed using Satterthwaite’s method (implemented in the “drop1” function in the “lmerTest” R package^29^). Based on Cook’s distance^30^ (implemented in the “check_outliers” function in the “performance” R package^31^) we excluded 2 observations that were classified as influential outliers in estimating Model 6. This did not change the direction of the relationship and slightly decreased the *F*-value. Throughout the manuscript, data are presented as mean (standard deviation) unless stated otherwise.

## Results

### Capturing Everyday Interactions

Social interactions were measured using RFID sensors assessing the physical proximity of participants (figure 2A). Badges were worn on 26 (SD 13) days out of potential 33 (SD 17) days by patients and 19 (SD 13) days out of 38 (SD 21) days by staff. This allowed a fine-grained observation of how patients and staff interacted with each other, and how these patterns changed on a scale of minutes (figure 2B and C). In total, 17 970 interactions were detected between 7 patients and 18 staff members (table 1). Figure 2D shows the distribution of interaction durations, which follows a long-tailed distribution, typically seen in this type of data.^14,15,32^

Note that in figure 2C, it is possible to recognize patterns of group activity, corresponding to meal preparation, lunch, and team meeting. When observing the temporal distribution of all recorded interactions, 1 notes distinct peaks around breakfast and lunchtime, as expected because on the Soteria ward patients and staff prepare and eat meals together. Toward the evening, the number of interactions decreases with a slight peak at dinnertime.

On average, patients spent 2.6 (SD 0.6) h per day with other people: 1.1 (SD 0.5) with staff and 1.9 (SD 0.8) h with other patients (table 1).

We tested how well objectively measured interactions correlated with subjectively reported quantity of social activity. There was no significant association between self-reported interaction time and measured relative interaction time (figure 2E, *F* = 0.01, *P* = .91). A large portion of the subjective responses was neutral (item 1:43%, item 2: 40%).

### Tracking Changes of Social Bonds Over Time

We first asked whether the amount of social interaction a patient changed during the course of the study. We did not observe a significant linear correlation between time and the relative amount of social interaction or the average duration spent in a single interaction.

**Fig. 3.**
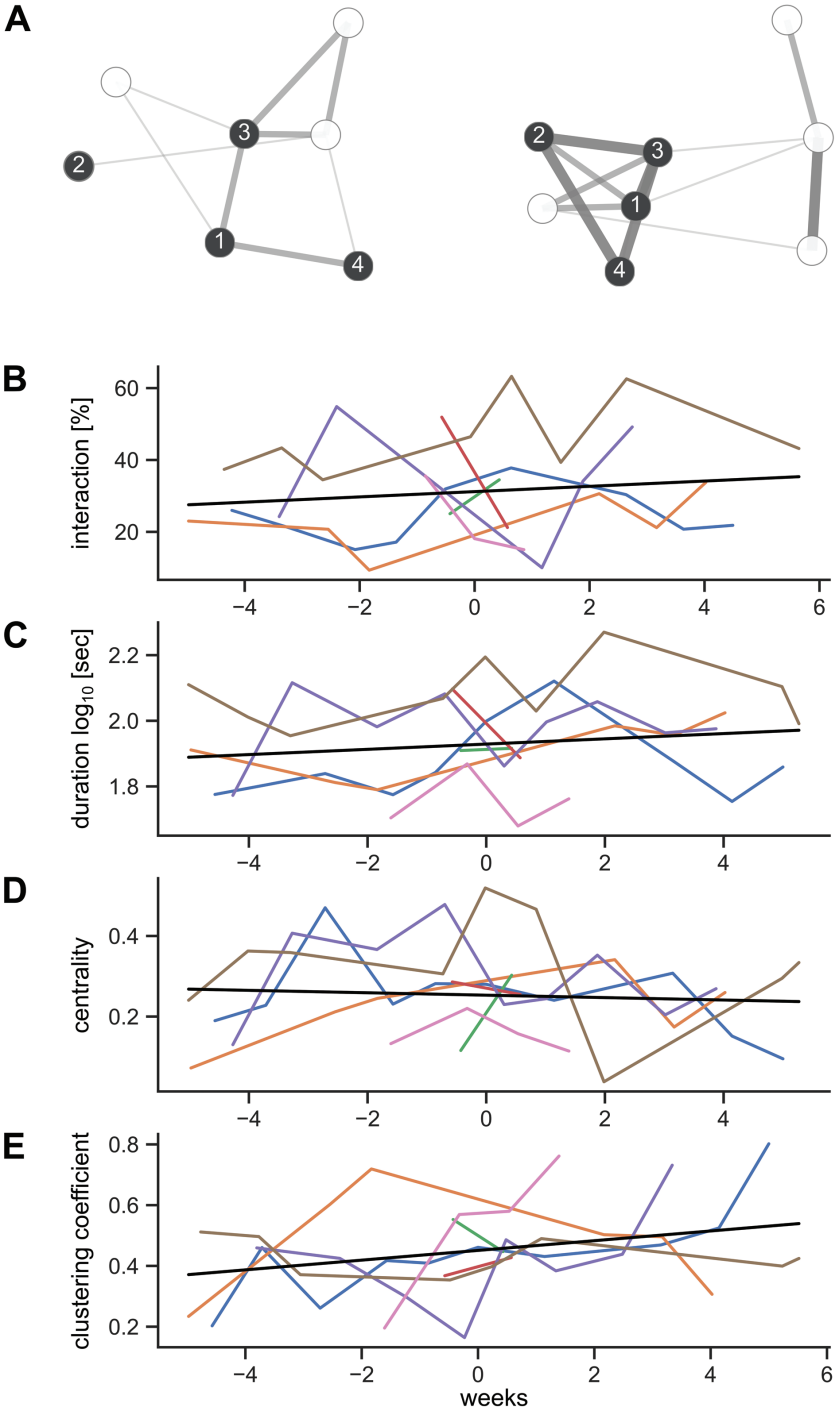
Parameters of social interaction over the course of the study. (A) Weighted, undirected graphs of 2 different representative days in the study. Patients (dark), staff (light). Edge thickness represents weight, ie, the common logarithm of the total duration of interaction between nodes. B-D. Mixed linear regression of relative interaction time (B), average interaction duration (C), eigenvector centrality (D), and clustering coefficient (E) against time.

To investigate how the relationship of the individual patient to the group evolves over time, we created weighted graphs of the interaction between participants for each day, where nodes represented participants and edge weights were derived from the total duration of interaction between 2 nodes on a given day. Thus, we calculated node attributes for each day and averaged them across the 6 days preceding and including the day of PANSS assessment (figure 3A). We computed weighted eigenvector centrality, which is a measure for the importance of a node in the network, and the weighted clustering coefficient, which measures how much the node and its connected neighbors cluster together.

While the eigenvector centrality did not show a linear association with time, there was an increase in the clustering coefficient over time (*F* = 4.18, *P* = .048), (figure 3B).

### Correlation Between Passively Measured Social Interaction and PANSS Score

**Fig. 4.**
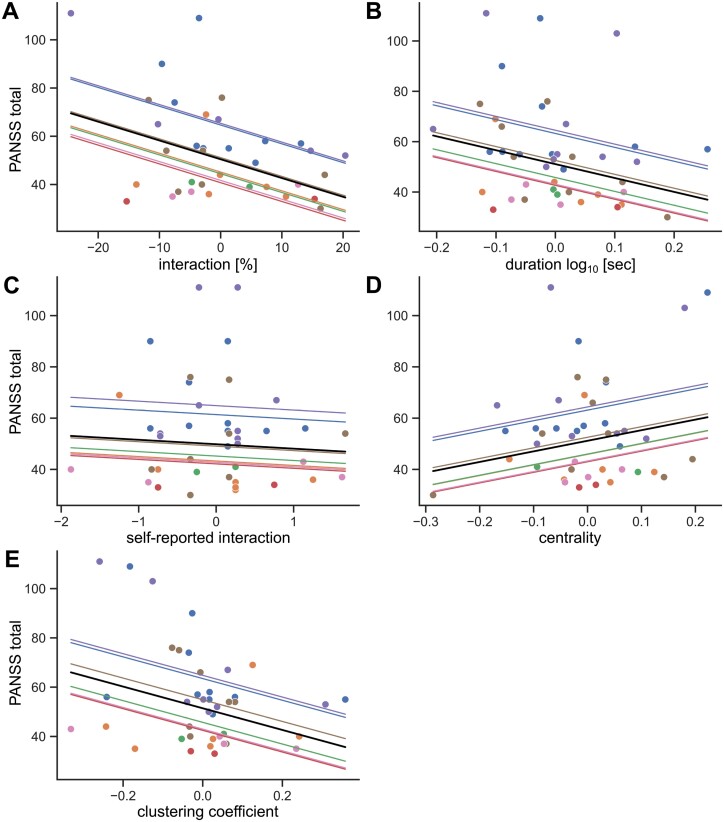
Association between parameters of social interaction and PANSS score. A–E. Mixed linear regression of PANSS total score and relative social interaction (A), interaction duration (B), self-reported social activity (C), eigenvector centrality (D), and clustering coefficient (E). Black line represents estimated fixed effect, shaded lines represent added random intercepts.

We further asked whether the amount of social interaction patients had during a given week was associated with the PANSS total score of that week (figure 4). The relative daily interaction time was negatively associated with PANSS total score (figure 4A, *F* = 5.64, *P* = .02). Likewise, the average interaction duration exhibited a negative correlation with PANSS total score (figure 4B, *F* = 4.13, *P* = .05).

Importantly, subjectively reported social interactions showed no association with PANSS scores (figure 4C, *F* = 0.23, *P* = .64).

Next, we investigated potential associations between graph theoretic measures and symptom severity. While eigenvector centrality, a measure of importance within a network, was not significantly related to PANSS scores (figure 4D, *F* = 2.36, *P* = .13), the clustering coefficient, which measures local integration, exhibited a negative correlation with PANSS total (figure 4E, *F* = 5.66, *P* = .02).

Adding olanzapine equivalent doses of daily antipsychotic agents at the beginning and end of the study as covariates of noninterest to our models did not change any of the significant findings (*P*_relative interaction_ = .001, *P*_mean duration_ = .04, *P*_clustering coefficient_ = .02). Interestingly, the associations between social interaction parameters and psychopathology were consistent in directionality across the positive, negative, and general psychopathology clusters (supplementary figure 3). Omitting staff interactions from the analysis did not change the directionality of the association; however, these associations were not significant (supplementary figure 4).

## Discussion

Here, we demonstrate the feasibility of detecting meaningful social interactions in a psychiatric hospital setting using passive sensor technologies. In addition, we show that several quantitative measures that capture individual and group-level aspects of social interaction were associated with patients’ total-PANSS scores in our sample. Being the first study in a psychiatric department setting, our study extends current knowledge on several axes.

First, the recorded data seem plausible: the long-tailed distribution of detected interaction durations indicate the wide and sparse distribution of longer interactions compared to an abundance of short interactions, similar to data previously observed at conferences^14,32^ and among university staff and students.^15^ Furthermore, the interaction data robustly reconstructs the known scheduled structure of social event at the Soteria ward such as breakfast, lunch, and dinnertime. Here, the distribution of interaction start times shows clear peaks around these events, supporting the validity of our measurements.

Patients spent ~30% of their day in active social engagement. While there is no similar data to compare, data from experience sampling (ESM) suggest that the daily active social engagement (ie, patients respond that they are both in company and interacting with that company) is usually lower in PSD patients.^33^ This could reflect the continuously offered opportunities for social activities on the ward. A definitive statement, however, is not possible, as the symptom burden in our sample was relatively low at study admission and the social motivation and social skills in our sample could be higher than in the average PSD patient. Nevertheless, it underscores the importance of offering a supportive setting, where the external factors contributing to social withdrawal, such as stigmatization, are removed. Secondly, subjectively rated duration of social interaction did not correlate with the objective RFID data. We suspect that recall bias as well as social desirability bias strongly influence the subjective ratings (most responses were “neutral”). Sensor data circumvent these sources of bias and offer high ecological validity: a study comparing contact diaries and RFID sensors in conference participants found large discrepancies between the 2 types of data with the diary method over-estimating the duration of contacts and underestimating the number of contacts <5 min^34^; another study, investigating social interactions in schizophrenia by means of smartphone sensors, found associations between ESM-reported social interaction and GPS and voice data.^35^ Thus, the investigation of social interaction as a symptom or disease mechanism might profit from objective quantitative measures in addition to retrospective self-reports.

Unlike most previous studies employing the RFID technology, which usually span a few days,^14,16^ we observed participants over the duration of several weeks, allowing for an analysis of changes in social interaction over time. Importantly, our combination of high-density mapping and graph analysis uncovered evidence for a key mechanism of social support: group integration. This was not explained by a simple increase in contacts over time as we did not find a linear trend in parameters that quantify the total amount of social interaction. Instead, we observed an increase in a marker that represents the integration into a social structure, namely the clustering coefficient. The fact that parameters of global interaction time remained stable across recovery might be explained by the high degree in which the Soteria engages patients usually right from the start of their stay in structured group activities. In contrast, our results hint toward a change in how patients interact and integrate in social groups, indicating that group patterns of social interaction contain relevant information about an individual. It is tempting to speculate that initially patients tend to have more “random” encounters with other people, but that their patterns change toward more structured and clustered interactions with a smaller set of known people.

In addition to providing a proof-of-concept for the objective assessment of social interactions, we observed an association between the social interaction pattern and symptom trajectories in our sample. The relative daily interaction time, the average interaction duration, and the clustering coefficient each were significantly associated with PANSS total. While not surprising, the negative association between relative daily interaction time and symptom severity confirms our most basic assumption that social interaction and disease severity are in fact related. The average interaction duration provides an additional simple measure of social interaction, which is less reliant on external factors. A tendency to stay longer in interactions could reflect a higher social motivation or improved skill at keeping the interaction going.

In addition, the clustering coefficient, a specific graph theoretic measure, was also associated with psychopathological symptoms. We take this metric, which is a measure of the connectedness of the other participants to which a patient is connected via social interactions, to indicate a form of integration into a group. This parameter had emerged in a recent cross-sectional study, which also used RFID sensors, as having the strongest correlation with workers’ mental health,^20^ highlighting the importance of social integration for mental well-being. Similarly, higher group integration was linked to lower PANSS total score in our sample.

### Limitations

#### Several Aspects of Our Work Require Special Consideration

First, our sample size is relatively small due to the complex requirements of our study that is couched within a routine clinical setting. However, the high sampling frequency and longitudinal design of the study enabled us to capture on average 26 days of RFID data per subject, building a solid database for each individual on which the analysis is based on. Future studies including more patients over longer periods are needed to confirm the results of our study.

Second, while nearly all staff participated in the study, not all of the patients on the ward during the study period did so. This leads to a potential underestimation of social interactions, as interactions with nonparticipating patients and staff were not recorded. Relatedly, we only recorded interactions between participants on weekdays, which excluded interactions with family, friends, and strangers at weekend or off-ward. While this certainly might also influence patients meaningfully, our results support the notion that we captured a large proportion of meaningful social interaction that is relevant for a patient’s recovery.

Third, we only measured the quantity and not the quality of social encounters. Based on the nature of the ward, we assume that most interactions are broadly positive for the participants. Future studies could aim to capture the emotional valence of interactions by event-triggered experience sampling.

Fourth, the particular composition of our sample and the specific setting limit generalizability. For example, our sample is limited to adolescents, early adults, and a specific disorder. Seventy-one percent were in their first episode of psychosis. Also, the mean PANSS total score at baseline was relatively low. This may be because some patients were receiving treatment before entering the study. Though we observed significant associations between interactions and changes in PANSS, our hypotheses and the applicability of our findings to other settings need to be tested in further studies.

#### Future Directions

Future studies should aim to recruit a larger set of patients and to minimize the number of people who are present but do not participate.

Having a larger dataset will also allow to correctly dynamically model changes in social ties and other network parameters. This type of high-resolution data could ideally be paired with outcome measures on a shorter timescale, such as symptoms and mood assessed by ESM, allowing to also classify the types of interaction by how patients appraise them. This will in turn allow to identify small-timescale patterns of social activity which might be especially helpful. Functional and structural magnetic resonance imaging has so far been helpful to identify neurobiological correlates of being socially excluded^36^ and even social network structure^37^ and promises to further improve understanding of the neurobiological mechanisms related to deficits in social interaction in PSD when coupled with RFID data.

## Conclusion

The data at hand clearly demonstrate the feasibility of conducting a longitudinal assessment of social interaction with wearable RFID devices in patients and staff. Our results suggest that markers of global interaction and especially measures that capture group integration are associated with symptomatology. This paves the way for future studies aiming for a more fine-grained, time-sensitive dissection of interaction patterns to the end of a better understanding of disease mechanisms as well as delivering personalized and targeted social interventions.

## Supplementary Material

Supplementary material is available at https://academic.oup.com/schizophreniabulletin/.

sbae032_suppl_Supplementary_Materials
