# Supplementary material for: Intensive Longitudinal Social Sensing in Patients With Psychosis Spectrum Disorders: An Exploratory Pilot Study
Source: Schizophr Bull. 2024 Mar 24;51(1):236–46. doi: 10.1093/schbul/sbae032 (PMC11661950; doi:10.1093/schbul/sbae032)
Supplement: sbae032_suppl_Supplementary_Materials [file sbae032_suppl_supplementary_materials.docx]

##
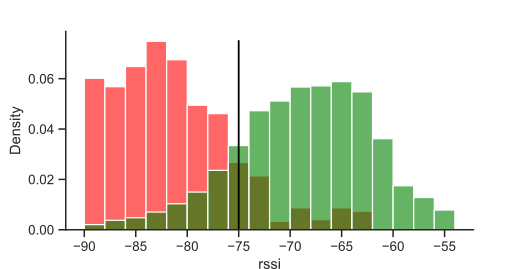


## Supplementary Figure 1. Determining the Received Signal Strength Indicator (RSSI) Threshold

Probability densities of RSSI values during situations of social interactions (green), and situations in which the experimenters were in the same room, not interacting (red). Data were obtained during a 50-minute long test run were some of the authors of the study simulated different daily situations. From the histogram, an RSSI value of -75 was deemed appropriate to separate hits (green) from misses (red).

##
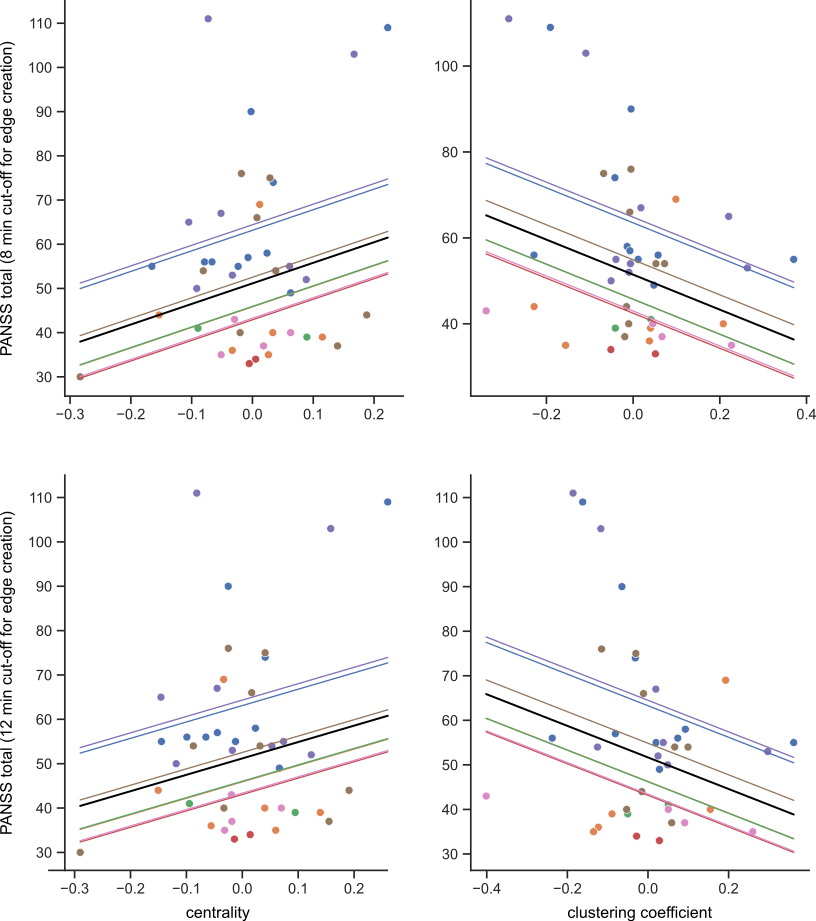


## Supplementary Figure 2. Determining the Edge Creation Threshold

Changing the cutoff value did not change the direction of the associations between parameters of social interaction and PANSS total with p_centrality >8 min_ = 0.10, p_clustering coefficient > 8 min_ = 0.03, p_centrality >12 min_ = 0.16, p_clustering coefficient > 12 min_ = 0.07.


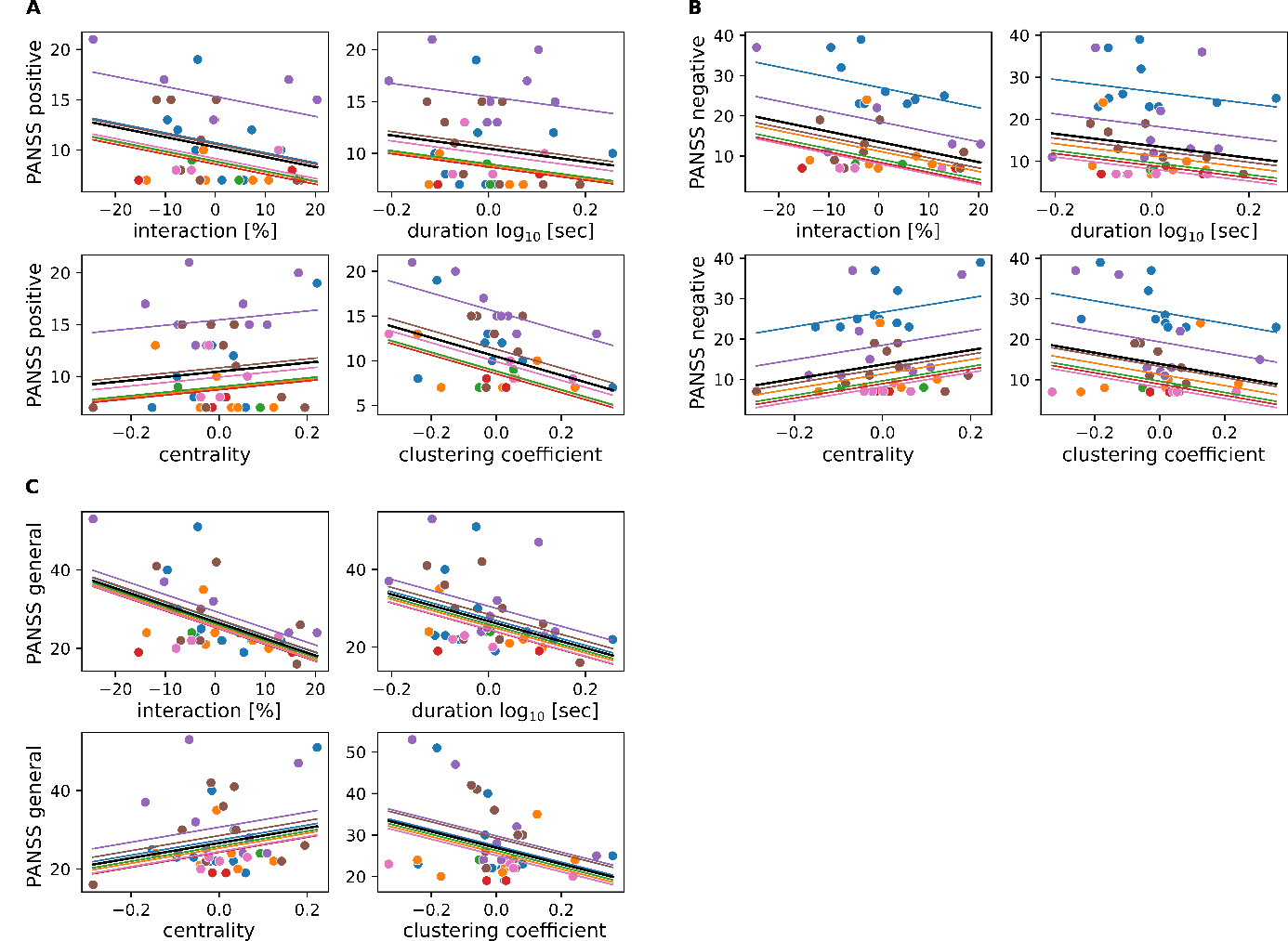


***Supplementary Figure 3. PANSS symptom clusters***

Associations between relative interaction (p=0.05), mean duration (p=0.21), centrality (p=0.39), and clustering coefficient (p=0.001) with the positive cluster (**A**).

Associations between relative interaction (p=0.01), mean duration (p=0.19), centrality (p=0.08), and clustering coefficient (p=0.06) with the negative cluster (**B**).

Associations between relative interaction (p=0.001), mean duration (p=0.01), centrality (p=0.15), and clustering coefficient (p=0.03) with the general psychopathology cluster (**C**).


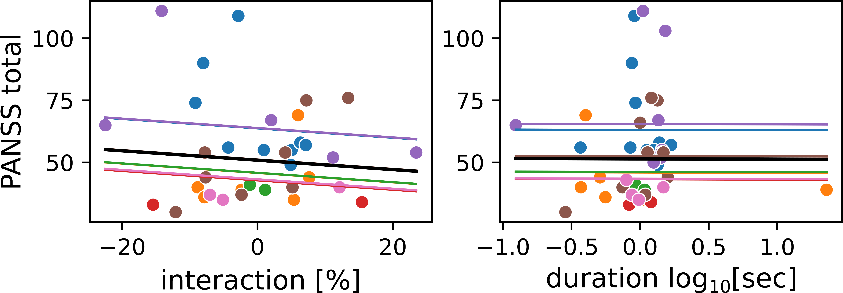


***Supplementary Figure 4. Omitting staff from the analysis***

When omitting interaction with staff from the analysis, there is no significant association between relative interaction (p = 0.54) or mean duration (p = 0.99) and PANSS total.
